# Magnetic resonance signs of intracranial hypertension in children: a retrospective case–control study

**DOI:** 10.1007/s00431-025-06025-8

**Published:** 2025-03-01

**Authors:** Luz Angela Moreno-Gómez, Daniel Quintero-Pulgarín, Oscar Mauricio Espitia Segura, Leidy Carolina Chiquiza-Garzón, Juan David Farfán-Albarracín, Cristina Lorena Ramírez-Sierra, Yenny Carolina Zuñiga-Zambrano, Leydi Alexandra Ceballos-Inga

**Affiliations:** 1 Department of Pediatric Radiology, HOMI Fundación Hospital Pediátrico la Misericordia, Bogotá, Colombia; 2 Qualitative and Quantitative Research Network in Child Neurology (RICCNeP), Bogotá, Colombia; 3 Department of Child Neurology, HOMI Fundación Hospital Pediátrico la Misericordia, Bogotá, Colombia; 4 School of Medicine, Universidad Nacional de Colombia, Bogotá, Colombia; 5 School of Medicine, Universidad de Antioquia, Medellín, Colombia; 6 Department of Child Neurology, Clínica Infantil Santa María del Lago - Clínica Colsanitas, Bogotá, Colombia

**Keywords:** Intracranial hypertension, Children, Magnetic resonance imaging, Opening pressure

## Abstract

The diagnostic accuracy of magnetic resonance (MRI) signs of intracranial hypertension (ICH) in children remains unclear. This study aims to assess how brain MRI signs of ICH correlate with cerebrospinal fluid opening pressure (CSFOP). A retrospective case–control study was conducted with 77 children aged 1 to 18 years, who underwent brain MRI and lumbar puncture with CSFOP measurement. A blinded radiologist evaluated key ICH signs, including distension of the perioptic subarachnoid space, optic nerve (ON) tortuosity, posterior scleral flattening, intraocular protrusion, and enhancement of the prelaminar ON, among others. Odds ratios, likelihood ratios, predictive values, sensitivity, specificity, and the area under the ROC curve (AUROC), were calculated in relation to CSFOP, adjusted for age and sex. The ICH group included 38 patients (49.3%) and the control group 39 (50.7%). The presence of four or more MRI signs of ICH provided the best diagnostic performance (specificity 92%, sensitivity 40%, AUROC 0.759) for identifying elevated CSFOP, particularly with intraocular protrusion of the prelaminar ON (*p* = 0.006, median CSFOP 49.6 cm H_2_O, *p* < .001) and papilledema (*p* = 0.042).

*Conclusion*: The presence of four or more MRI signs is associated with elevated CSFOP in children with non-structural ICH.

**Table:** 

**What Is Known:** *• The diagnostic accuracy of magnetic resonance imaging (MRI) signs of intracranial hypertension (ICH) in children remains unclear.* *• In children, there is no consensus about the most appropriate diagnostic criteria or robust evidence regarding the diagnostic performance of imaging signs for ICH.* **What Is New:** *• The presence of four or more MRI signs is associated with elevated cerebrospinal fluid opening pressure (CSFOP) in children with non-structural ICH.* *• Intraocular protrusion of the prelaminar ON, and papilledema are particularly relevant signs for identifying elevated CSFOP.*

**Supplementary Information:**

The online version contains supplementary material available at 10.1007/s00431-025-06025-8.

## Introduction

Pediatric intracranial hypertension (ICH) refers to elevated cerebrospinal fluid (CSF) pressure exceeding 28 cmH_2_O in children under sedation [1]. The condition can be classified as either “secondary”, resulting from identifiable causes such as hydrocephalus, neoplasms, thrombosis, or infections, or “primary” or “idiopathic” intracranial hypertension (IICH) with no structural causes [1, 2].

Diagnosis of IICH in adults is based on the accepted but expert opinion-based clinical criteria, including the modified Dandy criteria (1985) and the recently reviewed Friedman et al. criteria (2013), both of which include neuroimaging for ruling out structural causes of ICH and a normal composition of CSF [3].

Traditional clinical manifestations of ICH in children present diagnostic challenges, as papilledema—often a hallmark of ICH—is absent in up to 48% of pediatric IICH cases [2]. In children, there is no consensus about the most appropriate diagnostic criteria or robust evidence regarding the diagnostic performance of imaging signs for ICH [3, 4]. While elevated CSFOP by lumbar puncture is a well-known method to document ICH, accepted as part of the IICH criteria, its application may be restricted by technical or clinical limitations. In such cases, undiagnosed ICH particularly those from non-idiopathic but non-structural causes (e.g., inflammatory or infectious diseases) could lead to complications and long-term sequelae [2, 5].

Magnetic resonance imaging (MRI) is increasingly being proposed as a non-invasive diagnostic tool, where radiological signs related to ICH include distension of the perioptic subarachnoid space, optic nerve (ON) tortuosity, posterior scleral flattening, intraocular protrusion of the head of the ON, enhancement of the prelaminar portion of the ON, empty *sella turcica*, distension of Meckel’s cave, increased nuchal fat fold thickness, stenosis of transverse venous sinuses, and the temporal “thumb” sign [4].

The objective of this study was to evaluate the diagnostic performance of the MRI signs of ICH and its association with high CSFOP, including a broader definition that covers both idiopathic and secondary forms, but without structural lesions (i.e., space-occupying lesions) demonstrable in brain MRI, with standardized MRI signs.

## Materials and methods

### Research design

A retrospective case–control study was carried out at a single pediatric institution, HOMI Fundación Hospital Pediátrico la Misericordia in Bogotá, Colombia, from February 2017 to March 2023. Inclusion criteria were defined as children between 1 and 18 years with CSF opening pressure measured and MRI obtained less than 30 days apart, with images available for analysis, with non-acute diagnosis and with no interventions that could modify the pressure (Supplementary material Table 1). Exclusion criteria were the presence of intracranial mass-effect structural lesions on MRI. Cases were defined as children with CSFOP > 28 cmH_2_O due to any non-structural cause, and controls were children with CSFOP ≤ 28 cmH_2_O.

Lumbar punctures were no more than 1 month apart from the date of the MRI. Patients with MRI reports and/or medical record of space-occupying lesions, hydrocephalus, neuroinfection, obstruction, or other venous anomalies were omitted, to exclude patients with ICH of structural cause. MRI studies with incomplete sequences or with unacceptable artifacts also were excluded.

Lumbar puncture and CSFOP measurement were performed in operating rooms under general anesthesia with remifentanil and propofol, with patients in the left lateral decubitus position with flexed legs in all cases using a standardized semi-rigid pressure monitoring extension and recorded after 10 s of cerebrospinal fluid column stabilization, while normal pulsation was present and before sample collection, following the institutional protocol as previously reported [5].

### Imaging analysis

**Fig. 1:**
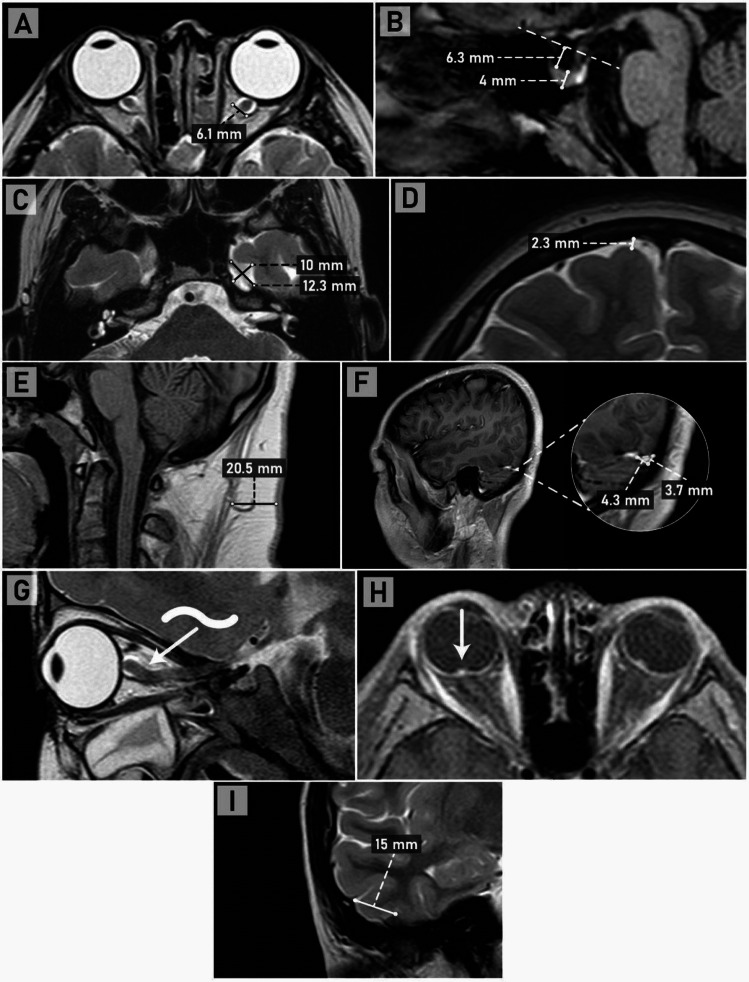
Interpretation of the MRI signs of ICH. **A** Bilateral increase in the subarachnoid space around the ON in axial T2 and flattening of the posterior sclera. **B** Sagittal T1 showing an “empty” (CSF occupied) *sella turcica* greater than 50%. **C** Axial T2 with increased amplitude of Meckel’s cave. **D** Subarachnoid space of the convexity. **E** Nuchal fat fold thickening, measured in sagittal T1. **F** Post-contrast 3D sagittal T1 image showing the measurement of two diameters of the left transverse sinus, triangular in shape, when comparing with a reference point 1.5 cm away from the torcula within the same sinus (not shown); there is a difference greater than 50% of the area, confirming stenosis. **G** Parasagittal T2 section of the orbit showing vertical tortuosity of the optic nerve. **H** Bilateral flattening of the posterior sclera, enhancement, and intraocular protrusion of the optic nerve head on axial contrast-enhanced T1 with fat saturation (arrow). **I** Positive temporal “thumb” sign on coronal T1

All images were acquired with a 1.5 Tesla Philips Multiva system, and the parameters are described in supplementary material. One radiologist with 20 years of experience in pediatric radiology and as a professor of neuroradiology evaluated the presence of the radiological signs. The radiologist was blinded for the CSFOP and for medical record data. Imaging data collection and measurements of all signs were standardized based on previous studies with pediatric patients (See supplementary material Table 2). Ten key MRI signs of ICH were measured: distension of the perioptic subarachnoid space; ON tortuosity; flattening of the posterior sclera; intraocular protrusion of the prelaminar portion or head of the ON; enhancement of the head of the ON; empty *sella turcica*; Meckel’s cave distension; temporal “thumb” sign; transverse venous sinus stenosis; and increased nuchal fat fold thickness (Fig. 1) [6–9].

The perioptic subarachnoid space was measured on axial T2 sequences, with a threshold of more than 4 mm. Flattening of the posterior sclera was evaluated qualitatively in axial T2 or sagittal T2 or T1 sequences, and enhancement and intraocular protrusion of the optic nerve head were evaluated on axial or sagittal contrast-enhanced T1 with fat saturation. Vertical tortuosity of the optic nerve was considered positive when it had the shape of the letter “S” on sagittal T1 or T2 [10]. Empty *sella turcica* was measured in sagittal T1, using a reference line drawn through the clinoid processes, through which a perpendicular measurement indicated the height of the pituitary gland. If more than 50% of the *sella* was occupied by CSF, the sign was positive [11, 12]. Enlargement of Meckel’s cave was measured on both axial and coronal T2 calculating the average diameter and considering that if the subarachnoid space of the convexity was increased, the increase in the size of Meckel’s cave could be related to volume loss of the brain parenchyma and therefore excluded for the analysis [8]. Nuchal fat fold thickening, as a surrogate for obesity, was measured in sagittal T1, with a cut-off point of 11 mm [10, 13]. Width and height of the triangular-shaped transverse sinuses were measured to calculate its area, comparing the area of a reference point 1.5 cm away from the torcula with the area in the smallest portion of the sinus; if a difference of more than 50% was found, the sign was considered positive for stenosis [13, 14]. The temporal “thumb” sign was evaluated on coronal T1, showing the remodeling of the posterior temporal skull base by the inferior temporal gyrus, with a base greater than 1 cm [15]. Detailed description of the measurement techniques is presented in the Supplementary material Table 2.

### Statistical analysis

Variables are presented as medians and interquartile ranges or frequencies and percentages as appropriate. The outcome variable was the CSFOP categorized as high (> 28 cmH_2_O) or normal (≤ 28 cmH_2_O). Univariate analysis for categorical variables was done with Fisher’s exact test or chi-square as appropriate, and the continuous variables with the Mann–Whitney test. Sensitivity, specificity, likelihood ratio (positive and negative test), and predictive values were estimated. To adjust for sex and age, given that are known CSFOP modifiers, penalized logistic regression model (Firth) was fitted for each independent variable (for the nuchal fat fold, also adjusted for the body mass index (BMI)); thus, the adjusted OR and the C statistic (ROC) were obtained for each sign. Firth regression was chosen to obtain more conservative estimates, as the patient sample is relatively small for a conventional logistic regression model [16]. As a sensitivity analysis, for assessing the robustness of the findings, opening pressure was also analyzed as a continuous variable to understand the magnitude of it among groups. Finally, it was evaluated whether the presence of multiple radiological characteristics simultaneously is different between cases and controls. Statistical analysis was performed with the Stata 18 software.

## Results

**Fig. 2:**
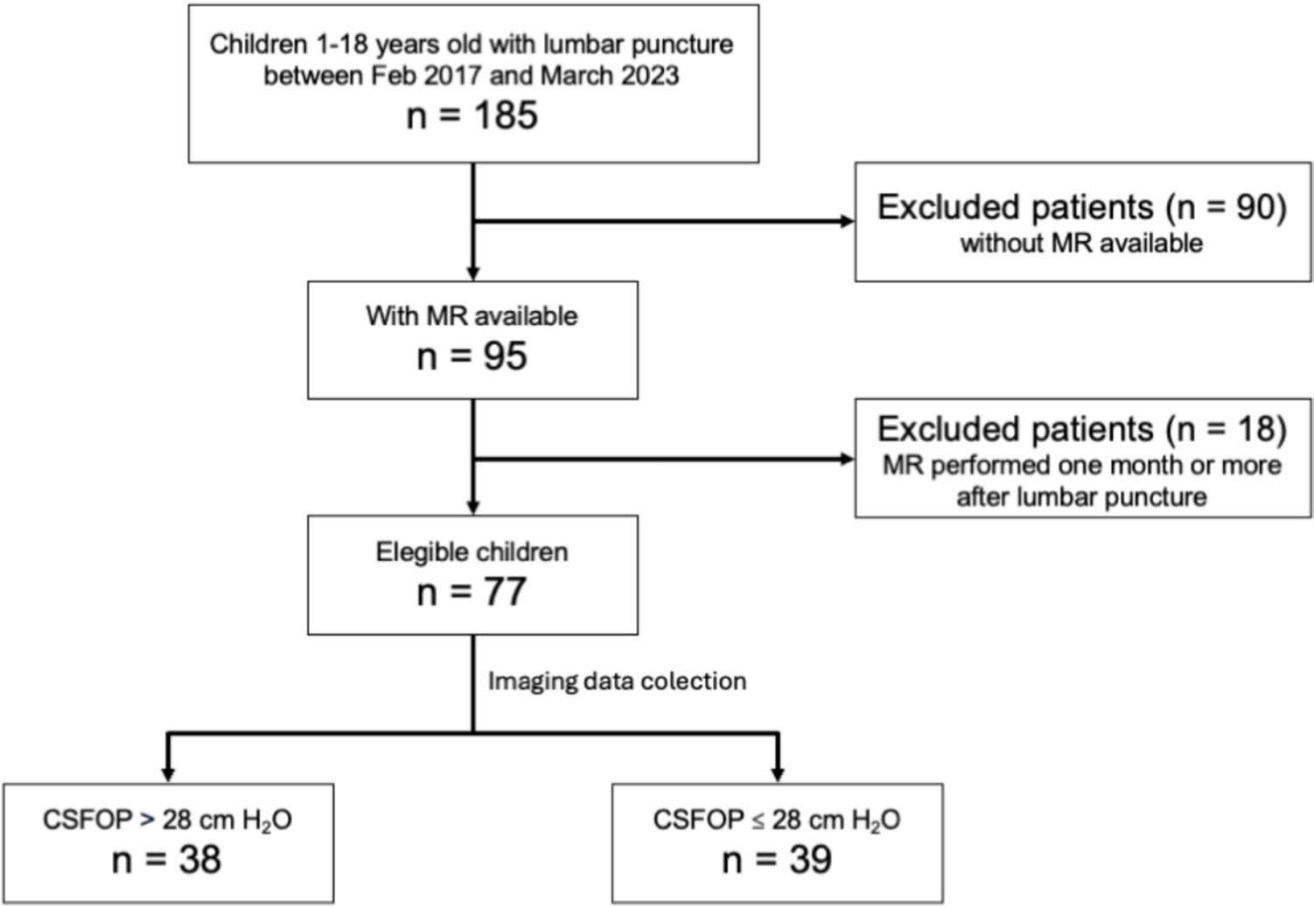
Distribution of study groups according to increased (> 28 cmH_2_O) or normal (28 ≤ cmH_2_O) CSFOP. CSFOP, cerebrospinal fluid opening pressure

A total of 185 patients underwent opening pressure measurement and had brain MRI performed during the study period. Of these, 95 MRI studies were available for review at the time of analysis and, after further selection, 77 patients with MRI performed less than 30 days apart from lumbar puncture were included. Ultimately, 88% (68/77) had MRIs completed within 10 days, and all were performed within 21 days of lumbar puncture (mean 3.26, median 3 days). Of the 77 patients analyzed, 38 were cases, while 39 were controls (Fig. 2).

The distribution of age and sex was homogeneous between the groups. Additional demographics and clinical characteristics are described in the Online Supplementary Data (Supplementary material Tables 3 and 4). The most frequent clinical features across all patients were headache (53%), seizures (32%), nausea (22%), and altered state of consciousness (18%). No statistically significant differences were observed between cases and controls for most clinical variables. Among the 60 patients (77.9%) who underwent fundoscopy, papilledema was observed in 15.6% (12/60). Of these, 83.3% (10/12) had elevated CSFOP, with a statistically significant difference between groups (*p* = 0.04) (Supplemental material Table 3).

When analyzing the diagnostic performance of the radiological signs of ICH, it was found that the intraocular protrusion of the head of the ON, ON head enhancement had a significantly greater prevalence in the ICH group. Additionally, posterior scleral flattening, transverse sinus stenosis, and Meckel’s cave distension were more frequent in cases compared to controls, although the difference was not significant (*p* < 0.1). Protrusion of the ON head was observed in nine patients with elevated opening pressure and only one patient without (*p* = 0.006), with a median CSFOP of 49.6 cmH_2_O (IQR 41.5–63; *p* < 0.001; Tables 1 and 2).

**Table 1:** Association between the presence or absence of ICH and each of the MRI signs

MRI sign No. (%)	High CSFOP		Normal CSFOP		
	Present	Absent	Present	Absent	*p*
ON tortuosity	13 (34.2)	25 (65.7)	8 (20.51)	31 (79.49)	0.177^b^
Posterior scleral flattening	16 (43.4)	21 (56,7)	9 (23)	30 (76.9)	0.061^b^
Intraocular ON head protrusion	9 (23.6)	29 (76.3)	1 (2.56)	38 (97.44)	0.006^a^
ON head enhancement	6 (22.2)	21 (77.7)	0 (0)	19 (100)	0.028^a^
Transverse sinus stenosis	8 (29.6)	19 (70.3)	2 (9.5)	19 (90.4)	0.089^a^
Temporal “thumb” sign	9 (24.32)	28 (75.6)	8 (20.5)	31 (79.4)	0.690^b^
Empty *sella*	7 (18.4)	31 (81.5)	3 (7.6)	36 (92.3)	0.161^a^
Nuchal fat fold thickening	6 (15.7)	32 (84.2)	2 (5.1)	37 (94.87)	0.125^a^
Perioptic subarachnoid space distension	18 (47.3)	20 (52.6)	15 (38.4)	24 (61.5)	0.430^b^
Meckel’s cave distension	30 (78.9)	8 (21)	23 (59)	16 (41)	0.059^b^

*CSFOP* cerebrospinal fluid opening pressure, *ON* optic nerve

^a^Fisher exact test

^b^Chi^2^

**Table 2:** Median of CSFOP and their relationship with the presence or absence of each MRI sign

MRI sign	Present	Absent	
	CSFOP Median cmH_2_O (IQR)	CSFOP Median cmH_2_O (IQR)	*p* value^a^
Optic nerve tortuosity	32.9 (21.5–39)	26.3 (17.5–33)	0.1077
Posterior scleral flattening	35.5 (25–46)	24.4 (17–31)	0.0051
ON head intraocular protrusion	49.6 (41.5–63)	24.9 (18–32.5)	0.0001
ON head enhancement	55.9 (50–63)	26.6 (18.7–34.2)	0.0001
Transverse sinus stenosis	38.7 (32.5–50)	27.3 (16–34)	0.0188
Temporal “thumb” sign	32.2 (19.5–49)	26.9 (19–34.5)	0.4904
Empty sella	38.7 (23–63)	26.5 (19–33)	0.0715
Nuchal fat fold thickening	41.9 (27–56)	26.5 (18–33)	0.0110
Perioptic subarachnoid space distension	31.8 (19–41)	25.3 (17.5–31.5)	0.1223
Meckel’s cave distension	29.8 (19–36)	24.5 (18.5–30)	0.1560

^a^Mann-Whitney test

*CSFOP* cerebrospinal fluid opening pressure, *IQR* interquartile range, *ON* optic nerve

Overall, 89.6% (69/77) of patients had at least one radiological sign of ICH, and 66.2% (51/77) had at least two signs. A significantly higher prevalence of radiological signs was observed in cases, particularly when three or more signs were identified. The optimal diagnostic performance was obtained with the presence of four or more radiological signs, with a sensitivity of 40% (95% CI 29–50) and a specificity of 92% (95% CI 86–98), with a discrimination capacity measured by AUROC of 0.759. Although a high specificity for ICH was obtained with the presence of five or more signs, this was accompanied by a notable decrease in sensitivity (Table 3 and supplementary material Table 5). Sensitivity analysis revealed a positive correlation between the magnitude of CSFOP and both the presence of each individual radiological sign and the cumulative number of signs observed.

**Table 3:** Diagnostic performance of the MRI signs of ICH

MRI sign	ICH	Sensitivity (%, IC95%)	Specificity (%, IC95%)	LR +	LR −	Adjusted OR (IC 95%)	*p*	AUROC^a^	PPV (%, IC95%)	NPV (%, IC95%)
ON tortuosity	13/38	34 (24–45)	80 (71–88)	1.66	0.83	2.22 (0.71–9.94)	0.170	0.720	61 (51–73)	55 (44–66)
Posterior scleral flattening	16/37	43 (32–54)	77 (68–86)	1.87	0.74	3.20 (1.07–9.63)	0.038	0.713	64 (53–75)	59 (48–70)
Intraocular protrusion of ON	9/38	24 (14–33)	97 (94–100)	9.24	0.78	7.58 (1.20–47.64)	0.031	0.725	90 (83 –97)	57 (46–68)
ON head enhancement	6/27	22 (10–34)	100 (100–100)	–	0.778	13.0 (0.67–250.28)	0.090	0.677	100 (100 –100)	47 (33 –62)
Transverse sinuses stenosis	8/27	30 (17–43)	91 (82–99)	3.11	0.78	2.85 (0.59–13.84)	0.194	0.664	80 (69–91)	50 (36–64)
Temporal thumb sign	9/37	24 (15–34)	80 (70–89)	1.19	0.95	1.05 (0.34–3.22)	0.928	0.676	53 (42–64)	52 (41–64)
Empty sella	7/38	18 (10–27)	92 (86–98)	2.40	0.88	2.48 (0.59–10.41)	0.215	0.696	70 (60–80)	54 (42–65)
Nuchal fat fold thickening	6/38	16 (8–24)	95 (90–100)	3.08	0.89	3.53 (0.58–21.33)	0.170	0.672	75 (65–85)	54 (42–65)
Perioptic subarachnoid space distension	18/38	47 (36–59)	62 (51–72)	1.23	0.86	1,37 (0.51–3.66)	0.533	0.689	54 (43–66)	54 (43–66)
Meckel’s cave distension	30/38	79 (70–88)	41 (30–52)	1.34	0.51	1.83 (0.64–5.24)	0.263	0.687	57 (45–68)	67 (56–77)
Number of signs present										
1 or more	36/38	95 (90–100)	15 (7–23)	1,12	0,34	1.88 (0.38–9.21)	0.436	0.688	52 (41–63)	75 (65–85)
2 or more	27/38	71 (61–81)	39 (28–49)	1.16	0.75	1.17 (0.43–3.22)	0.750	0.686	53 (42–64)	58 (47–69)
3 or more	18/38	47 (36–59)	74 (65–84)	1,85	0,71	2.22 (0.81–6.05)	0.121	0.700	64 (54–75)	59 (48–70)
4 or more	15/38	40 (29–50)	92 (86–98)	5,13	0,66	8.02 (2.04–31.46)	0.003	0.759	83 (75–92)	61 (50–72)
5 or more	9/38	24 (14–33)	97 (94–100)	9.24	0.78	9.21 (1.39–61.04)	0.021	0.733	90 (83–97)	57 (46–68)

*ON* optic nerve, *ICH* intracranial hypertension, *LR* likelihood ratio, *OR* odds ratio, *Adjusted OR* adjusted for age and sex (and for BMI in nuchal fat fold thickening), *AUROC* area under the ROC curve, *PPV* positive predictive value, *NPV* negative predictive value

^a^AUROC after Firth penalized logistic regression model for sex, age and BMI in the nuchal fat fold thickening

## Discussion

This study investigated a cohort of pediatric patients to analyze the utility of radiological signs on brain MRI in predicting elevated CSF opening pressure. The findings suggest that the presence of four or more radiological signs is associated with greater diagnostic performance, while the presence of one or two signs could represent incidental findings, even in individuals with normal CSFOP.

Most studies correlating clinical and imaging variables in ICH use the established clinical criteria for the diagnosis of IICH as their reference standard, such as Friedman’s criteria and Dandy’s criteria. However, these are based on expert consensus rather than robust evidence-based data [2, 3]. However, elevated ICH, and thus high CSFOP, has been observed even in children who do not meet the clinical criteria for IICH, and without the presence of lesions in neuroimaging, such as in patients with neuroinfection or Guillain-Barré syndrome [17]. Based on the hypothesis that imaging changes have a more direct and greater relationship with CSFOP elevation compared to clinical manifestations, it appears necessary to investigate to what extent this association is satisfied.

In this study, the most commonly reported symptom was headache, consistent with available data of ICH studies in children; however, it was also the main symptom in patients without ICH [12], highlighting the fact that this is the most frequent indication for both MRI and opening pressure measure and that it has a low value in predicting elevation in opening pressure, particularly in children over 10 years of age [5]. Unlike other studies [2], predominance by sex or a higher frequency of obesity was not found in this study; this might be partially secondary to the low prevalence of obesity in this cohort. Papilledema was more frequent in the high CSFOP group, with a statistically significant difference; however, its absence cannot rule out the diagnosis of ICH reliably, as demonstrated in this study and previous research [18]. In fact, ophthalmologic evaluation, if available, often fails to detect papilledema in up to half of cases of ICH particularly in early stages [2]. The low prevalence of papilledema in this cohort highlights the need for alternative diagnostic markers. This limitation is especially relevant in settings where access to ophthalmologic evaluation is restricted or delayed.

Radiologic signs for ICH have been studied recently with several measures demonstrating high interrater reliability [19]. This reliability allowed for a single radiologist —who was blinded to clinical data— to analyze the imaging in this study. Ten standardized radiological signs were evaluated, and consistent with findings in other reports, it is common that even in patients without high CSFOP, at least one or two signs of ICH are found by MRI [9]. Most individual signs were observed more frequently in cases; however, only the intraocular protrusion of the head of the ON and ON head enhancement were significantly more prevalent among cases. However, the combination of four or more signs of ICH by MRI is associated with better diagnostic performance and with a better discrimination capacity as measured by the AUROC. Moreover, in the sensitivity analysis, the median CSFOP is significantly higher in the presence of at least four MRI signs. Among clinical signs, papilledema was the only one significantly more frequent in cases compared to the control group, which is related to the anatomical alterations reflected in the radiological signs with the best diagnostic performance, namely, flattening of the posterior sclera and intraocular protrusion of the optic nerve. This is in accordance with the known relationship between clinical papilledema and ICH, widely reported in the literature [10, 20].

These results provide a preliminary exploration of the diagnostic value of radiological signs for detecting high CSFOP in children. Strengths of this study include the relatively high sample size (*n* = 77) and multivariate adjustments made for covariates known as CSFOP modifiers, such as age and sex, with the use of penalized logistic regression (Firth), as the fact that lumbar punctures are performed as indicated by clinical indications, rather than research purposes, reflecting real-world clinical scenarios.

Among the study limitations is the retrospective design precluding the possibility for a shorter time between lumbar punctures and MRIs, which might miss acute changes; however, patients had chronic conditions as indication for lumbar puncture and while a long interval from opening pressure to MRI can induce bias, it was addressed including only patients with no intervention between studies, as reported elsewhere [21]. An additional limitation of this study is the low observed prevalence of papilledema; however, this might reflect real-world practice in developing countries where availability of ophthalmological evaluation is limited, and proxy measures should be used. Additional study designs are required based on the possible economic and clinical impact of introducing MRI into the standard multimodal diagnostic approach, which could have the advantage of detecting more cases allowing an earlier treatment; also, a prospective design might include patients with shorter intervals between LP and MRI.

## Conclusions

Our results suggest that brain MRI has a high specificity in the diagnostic workup of children with suspected high CSFOP, and therefore ICH. A high CSFOP should be considered in children with four or more MRI signs, especially if protrusion and enhancement of the head of the ON and clinical papilledema are included.

The incidental finding of one or a few radiological signs of ICH is common; thus, no individual radiological sign is useful. The clinical and economic impact of introducing MRI in the diagnostic workup of ICH should be assessed in specific studies.

## Supplementary Information

Below is the link to the electronic supplementary material.

Supplementary file1 (DOCX 2654 kb)

## Data Availability

No datasets were generated or analysed during the current study.
