# Supplementary material for: Magnetic resonance signs of intracranial hypertension in children: a retrospective case–control study
Source: Eur J Pediatr. 2025 Mar 1;184(3):218. doi: 10.1007/s00431-025-06025-8 (PMC11872764; doi:10.1007/s00431-025-06025-8)
Supplement: Supplementary file 1 — Supplementary file1 (DOCX 2654 kb) [file 431_2025_6025_MOESM1_ESM.docx]

**SUPPLEMENTARY MATERIAL**

**Supplementary Table 1.** Children’s diagnosis included in the study.

| **Diagnosis** | **Frequency** | **Percent (%)** | **CRF**^1^ |
| --- | --- | --- | --- |
| Ataxia | 1 | 1.30 | 1.30 |
| Headache | 34 | 44.16 | 45.45 |
| Cranial Nerve Alterations | 1 | 1.30 | 46.75 |
| Autoimmune diseases | 13 | 16.88 | 63.64 |
| Epilepsy | 21 | 27.27 | 90.91 |
| Other | 7 | 9.09 | 100.00 |
| Total | 77 | 100 |  |
| CRF: Cumulative relative frequency | | | |

**Supplementary Table 2.** MR signs operational definition.

| **MR sign** | **Definition** |
| --- | --- |
| **ON vertical tortuosity** | “S” morphology in sagittal T1 or T2. [14] |
| **Posterior scleral flattening** | Flattening of the posterior border of the eyeball. [14] |
| **Intraocular ON head protrusion** | Protrusion of the optic nerve into the eyeball. [14] |
| **ON head enhancement** | Enhancement of the prelaminar portion of the ON. [14] |
| **Transverse sinus stenosis** | First, in contrast- enhanced T1 or MR venography orthogonal measurements of width and height measured at 1.5 cm lateral to the central point of the torcula as a reference point. The point of greatest stenosis, or the apex of the ascending curve of the transverse sinus, proximal to the sigmoid sinus were measured. The product between them was divided by 2 to obtain an area of the triangle in mm2 and a decrease of more than 50% of its area compared to the reference point was determined as positive. [16] |
| **Temporal “thumb” sign** | Remodeling of the posterior temporal bone by the inferior temporal gyrus in coronal T2, with a depth of 1 cm or more. [17] |
| **Empty *sella turcica*** | Occupation of the sella by the hypophysis < 50% in sagittal T1 or T2, quantitatively comparing the length of its depth from a reference line between the anterior and posterior clinoid processes, with the height of the gland in the same axis perpendicular to the reference line. [19] |
| **Nuchal fat fold thickening** | Anteroposterior diameter in sagittal T1, at the level of the synchondrosis of the body of C2. Thickness > 11 mm was considered positive. [12] |
| **Perioptic subarachnoid space distension** | Transverse diameter in axial T2 between the internal surfaces of the arachnoid > 4 mm, measured 5 mm posterior to the posterior border of the sclera. [12] |
| **Meckel’s cave distension** | Average of the maximum perpendicular diameters measured in axial and coronal T2. Was considered positive when the coronal average was > 5 mm and/or in the axial plane > 8 mm. [14,15] |

**Supplementary Table 3.** Demographic and anthropometric characteristics of patients with and without ICH. ICH, Intracranial hypertension. IQR, interquartile range.

| **Characteristic** | **N** | **All**,  N=77 (100%)*^1^* | **Without ICH**  N=39 (51%)*^1^* | **With ICH**  N=38 (49%)*^1^* | **p-value***^2^* |
| --- | --- | --- | --- | --- | --- |
| **Age (months)** | 77 | 140  (65-181) | 103   (47.5-172) | 144.5  (98.5-191) | 0.057 |
| **Sex** | 77 |  |  |  | 0.2 |
| Female |  | 45 (58%) | 26 (67%) | 19 (50%) |  |
| Male |  | 32 (42%) | 13 (33%) | 19 (50%) |  |
| **Weight (kg)** | 76 | 40.5 (17-54) | 34.0  (14-53.3) | 46.6  (23.5-54) | 0.10 |
| **Height (cm)** | 71 | 150  (119-158) | 145  (103.5-157.5) | 152  (127-158.3) | 0.14 |
| **Body mass index (kg/m²)** | 71 | 18.7  (16,1-22.7) | 18.4  (16.5-22.2) | 19.2  (15.8-22.9) | 0.5 |
| **Obesity** | 77 | 8 (10%) | 5 (13%) | 3 (7.9%) | 0.7 |
| *^1^* Median (IQR); n (%) | | | | | |
| *^2^* Mann-Whitney test; Pearson’s Chi-squared test; Fisher’s exact test | | | | | |

**Supplementary Table 4.** Signs and symptoms in patients with or without ICH. ICH, Intracranial hypertension.

| **Sign/Symptom** | **N** | **All**,  N=77  (100%)*^1^* | **Without ICH**,  N=39  (51%)*^1^* | **With ICH**, N=38  (49%)*^1^* | **p-value***^2^* |
| --- | --- | --- | --- | --- | --- |
| **Headache** | 77 |  |  |  | 0.3 |
| No |  | 35 (45%) | 15 (38%) | 20 (53%) |  |
| Yes |  | 41 (53%) | 23 (59%) | 18 (47%) |  |
| No data |  | 1 (1.3%) | 1 (2.6%) | 0 (0%) |  |
| **Diplopia** | 77 |  |  |  | 0.030 |
| No |  | 70 (91%) | 34 (87%) | 36 (95%) |  |
| Yes |  | 2 (2.6%) | 0 (0%) | 2 (5.3%) |  |
| No data |  | 5 (6.5%) | 5 (13%) | 0 (0%) |  |
| **Blurred vision** | 77 |  |  |  | 0.2 |
| No |  | 63 (82%) | 30 (77%) | 33 (87%) |  |
| Yes |  | 11 (14%) | 6 (15%) | 5 (13%) |  |
| No data |  | 3 (3.9%) | 3 (7.7%) | 0 (0%) |  |
| **VI cranial nerve palsy** | 77 |  |  |  | >0.9 |
| No |  | 72 (94%) | 37 (95%) | 35 (92%) |  |
| Yes |  | 5 (6.5%) | 2 (5.1%) | 3 (7.9%) |  |
| **Other cranial nerve palsy** | 77 |  |  |  | >0.9 |
| No |  | 70 (91%) | 36 (92%) | 34 (89%) |  |
| Yes |  | 7 (9.1%) | 3 (7.7%) | 4 (11%) |  |
| **Vertigo** | 77 |  |  |  | 0.2 |
| No |  | 63 (82%) | 30 (77%) | 33 (87%) |  |
| Yes |  | 11 (14%) | 6 (15%) | 5 (13%) |  |
| No data |  | 3 (3.9%) | 3 (7.7%) | 0 (0%) |  |
| **Tinnitus** | 77 |  |  |  | 0.2 |
| No |  | 71 (92%) | 34 (87%) | 37 (97%) |  |
| Yes |  | 3 (3.9%) | 2 (5.1%) | 1 (2.6%) |  |
| No data |  | 3 (3.9%) | 3 (7,7%) | 0 (0%) |  |
| **Nausea** | 77 |  |  |  | 0.4 |
| No |  | 59 (77%) | 28 (72%) | 31 (82%) |  |
| Yes |  | 17 (22%) | 10 (26%) | 7 (18%) |  |
| No data |  | 1 (1.3%) | 1 (2.6%) | 0 (0%) |  |
| **Emesis** | 77 |  |  |  | 0.4 |
| No |  | 65 (84%) | 31 (79%) | 34 (89%) |  |
| Yes |  | 11 (14%) | 7 (18%) | 4 (11%) |  |
| No data |  | 1 (1.3%) | 1 (2.6%) | 0 (0%) |  |
| **State of consciousness alteration** | 77 |  |  |  | 0.8 |
| No |  | 63 (82%) | 31 (79%) | 32 (84%) |  |
| Yes |  | 14 (18%) | 8 (21%) | 6 (16%) |  |
| **Epileptic seizures** | 77 |  |  |  | 0.2 |
| No |  | 52 (68%) | 23 (59%) | 29 (76%) |  |
| Yes |  | 25 (32%) | 16 (41%) | 9 (24%) |  |
| **Ataxia** | 77 |  |  |  | 0.4 |
| No |  | 70 (91%) | 37 (95%) | 33 (87%) |  |
| Yes |  | 7 (9.1%) | 2 (5.1%) | 5 (13%) |  |
| **Papilledema** | 77 |  |  |  | 0.042 |
| No |  | 48 (62%) | 26 (67%) | 22 (58%) |  |
| Fundoscopy not performed |  | 17 (22%) | 11 (28%) | 6 (16%) |  |
| Bilateral |  | 10 (13%) | 1 (2,6%) | 9 (24%) |  |
| Unilateral |  | 2 (2.6%) | 1 (2,6%) | 1 (2.6%) |  |
| **Campimetric alterations** | 77 |  |  |  | <0.001 |
| Not performed |  | 58 (77%) | 38 (97%) | 1 (2.6%) |  |
| Normal |  | 15 (19%) | 1 (2.6%) | 14 (37%) |  |
| Central bilateral defect |  | 1 (1.3%) | 0 (0%) | 1 (2.6%) |  |
| Other |  | 2 (2.6%) | 0 (0%) | 2 (5.3%) |  |
| *^1^* n (%) | | | | | |
| *^2^* Mann-Whitney test; Pearson’s Chi-squared test; Fisher’s exact test | | | | | |

**Supplementary Table 5.** Medians of CSFOP with regard of the number of MR signs found

| **Number of MR signs** | **OCSFP (median (cmH2O), IRQ)** |
| --- | --- |
| At least 1 | 28.7 (19 - 36) |
| At least 2 | 29.8 (19 - 37) |
| At least 3 | 34.4 (23 - 44) |
| At least 4 | 41.8 (31 - 55) |
| At least 5 | 50 (36 - 63) |

MR, magnetic resonance. CSFOP, Cerebrospinal fluid opening pressure, IQR, Interquartile range.

**MRI parameters and sequence acquisition**

The technical characteristics of MRI sequence acquisition included were:

- Axial T2_TSE: TE: 110, TR 8148, FOV 231 x 185 x 147 mm, voxel 0.599 x 0.75 x 3 mm, matrix 384 x 239 x 37 slices, gap 1 mm.

- Coronal T2_TSE: TE 110 TR 8164, FOV 200 x 200 x 122 mm, voxel 0.6 x 1.1 x 3 mm, matrix 322 x 178 x 36 slices, gap 0.4 mm.

- Sagittal T2_TSE (Orbits MRI): TE 100, TR 2097, FOV 140 x 140 x 52 mm, voxel 0.48 x 0.7 x 2.5 mm, matrix 292 x 198 x 19 slices, gap 0.25 mm

- Sagittal T1_3D simple: TE 4.3, TR 8.5, FOV 249 x 249 x 170 mm, voxel 1 x 1 x 2 mm, matrix 248 x 232 x 85 slices, gap 0 mm.

- Sagittal T1_3D contrast-enhanced: TE 4.3, TR 8.5, FOV 249 x 249 x 170 mm, voxel 1 x 1 x 2 mm, matrix 248 x 232 x 85 slices, gap 0 mm.

- Axial T1 contrast-enhanced: TE 14, TR 638, FOV 230 x 216 x 149 mm, voxel 0.88 x 1.6 x 5 mm, matrix 280 x 134 x 25 slices, gap 1 mm.

- Coronal T1 contrast-enhanced: TE 3.9, TR 394, FOV 200 x 200 x 158 mm, voxel 0.66 x 1.11 x 4 mm, matrix 304 x 180 x 36 slices, gap 0.4 mm.
